# Implementing the Design of Experiments (DoE) Concept into the Development Phase of Orodispersible Minitablets (ODMTs) Containing Melatonin

**DOI:** 10.1208/s12249-021-02185-6

**Published:** 2022-01-20

**Authors:** Arkadiusz Hejduk, Michał Teżyk, Emilia Jakubowska, Klaudia Krüger, Janina Lulek

**Affiliations:** 1 Chair and Department of Pharmaceutical Technology, Faculty of Pharmacy, Poznan University of Medical Sciences, 6 Grunwaldzka Street, 60-780 Poznan, Poland; 2 LEK-AM Pharmaceutical Company Ltd, 14A Ostrzykowizna Street, 05-170 Zakroczym, Poland

**Keywords:** orodispersible minitablets, Plackett-Burman, design of experiments, design space, melatonin

## Abstract

Development of orodispersible minitablets (ODMTs) requires consideration of aspects related to small dimensions, while ensuring short disintegration time with sufficient mechanical stability. In order to meet these and other critical quality attributes (CQAs), quality by design is encouraged. According to this approach, formulation and compression process factors were systematically studied using design of experiments (Plackett-Burman for screening purposes, full and fractional factorial design for in-depth characterization) to understand their influence on CQAs of orodispersible minitablets containing melatonin. Mathematical models describing the relationships between processing variables and attributes such as resistance to crushing and disintegration time were successfully developed, characterized by high coefficients of determination (*R*^2^_adj_ = 0.90–0.97) and prediction errors in the range (+2.4 to −10.8%). In conclusion, based on these models, the design space was created for melatonin ODMTs, ensuring the product’s quality and process robustness. Moreover, the study demonstrated the suitability of texture analysis as an alternative to compendial measurement methods of resistance to crushing and disintegration time.

**Supplementary Information:**

The online version contains supplementary material available at 10.1208/s12249-021-02185-6.

**Figure:**
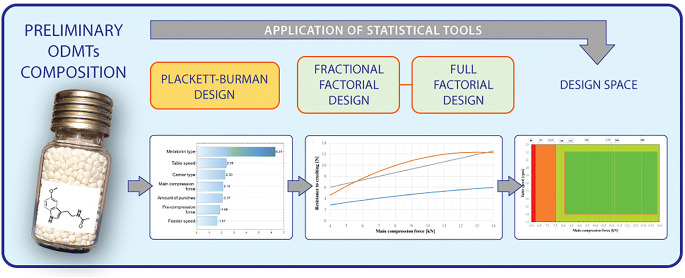
Graphical abstract

## INTRODUCTION

Orodispersible minitablets (ODMTs) are relatively novel dosage forms mainly intended for pediatric or geriatric patients (1). Due to the small diameter (≤ 3 mm) and fast disintegration time in the oral cavity, minitablets (MT) might be applied in many cases where swallowing difficulties do not allow the intake of standard oral solid forms such as tablets or capsules. However, ODMT production technology requires an understanding of all variables that affect product and process quality, especially considering the specific challenges when compared to conventional tablets. For instance, in the filling process of small die orifices, the effects of pressure gradients and air entrainment are more pronounced than for larger diameters (2), and minitablet tooling is more sensitive to abrasion and damage than regular punch tips (1). As small absolute weight variations might result in significant potency differences between units, tolerance for non-uniform die filling and mass variability is also restricted for minitablets (3). Another challenge posed by their dimensions is the assurance of content uniformity and the difficulties in relating this parameter to drug substance particle size and concentration in the blend (4). Besides manufacturing aspects, minitablets often require fine-tuning of analytical tools, as testing equipment suitable for conventional tablets might not be sensitive enough for the determination of mechanical properties or disintegration time of dosage forms below 3 mm (4, 5).

Several examples of development and characterization of minitablets with various release types have been described, e.g., (6–15). However, to the authors’ knowledge, reports of comprehensive approach to minitablets’ formulation and process optimization in an industrially relevant setting are lacking in the publicly available literature, especially in the topic of ODMTs. While there are several valuable in-depth studies on formulation and compression of minitablets, they mostly focus on select narrow aspects, often employing placebo blends (2, 16–20) or equipment restricted to laboratory scale (eccentric tablet presses (4, 16, 17, 21) or compaction simulators (18, 19, 22)). For the majority of the aforementioned research, the main focus has been on formulation characteristics, namely the choice of excipients type or grade, as well as particle sizes in the context of blend flow properties. In one of the first works on ODMTs, Stoltenberg and Breitkreutz compared the performance of different co-processed excipients based on mannitol in terms of minitablet attributes at compression forces of 3–10 kN. All the blends displayed acceptable flowability without observable influence on tablet mass and content uniformity. Based on crushing strength values, friability, and wetting time, Ludiflash was identified as the optimal co-processed excipient for ODMTs, while Pearlitol Flash or Prosolv failed to produce minitablets with sufficient mechanical strength (1). In a rare example of API evaluation, Mitra et al. studied the influence of two types of acetaminophen with differing morphologies and particle sizes at different drug loads. Increasing the API particle size from 30 to 170 μm and reducing its concentration in the blend from 26.7 to 6.7% resulted in worse content uniformity, although lower drug load permitted ODMTs with higher tensile strength (4).

Several studies have been carried out where formulation characteristics like flow properties or particle size are evaluated along with processing variables. For example, Hagen et al. compared different sized fractions of mannitol in interactive mixtures with sodium salicylate, as well as mixer type and mixing time in terms of blend homogeneity and minitablets’ weight and content uniformity (22). An important area of research has been the investigation of feeding and die filling in relation to minitablet weight and its variation. Zhao et al. found no difference between gravity feeder and force feeder applied to filling 1.7 mm die orifices on a single station tablet press. Instead, mass variability was influenced by the MCC grade and particle size, governing its flow properties. However, despite their best flow, excessively large granules (fraction collected from 1 mm sieve) resulted in higher variation of minitablet weight and hardness (16). This confirmed the rule postulated by Flemming and Mielck that particles must not be larger than 1/3 of the diameter of an orifice to flow successfully through it (23), practically indicating the critical size for powders or granules to be compressed into minitablets. Regarding feeder type, contradictory results are described by Kurashima et al., who also considered the tip and orifice position for a 2.3 mm, 12-tip tooling in a rotary tablet press. The authors studied the effect of open or force feeder and rotational speed (20–60 rpm) for lactose- or mannitol-based granulations with different particle sizes (24). Minitablet die filling conditions have also been studied with respect to different orifice sizes. Kachrimanis et al. investigated the flow rates of blends with various densities and particle sizes through orifices of different diameters (2–4 mm) and lengths (0.5–1.5 cm), corresponding to diverse die thicknesses (2). In a case study described by Rumondor et al., the effect of poor blend flow on minitablet weight variation was more pronounced for 2 mm than for 3-mm diameter, whereas turret and feeder rotation speeds did not have a significant influence (3). In another report, Goh et al. investigated minitablet weight variability within one compression cycle on a rotary press and between several cycles. Granules with different flow properties were fed with a force feeder into two die sizes: 1.8 mm (9-tip tooling) and 3 mm (8 tips). Considering intracycle variability, smaller dies were found to be more difficult to fill uniformly, as residual entrapped air was not released efficiently and back pressure hindered feed entry, which resulted in more cohesive powders arching over orifice. The authors concluded that for larger dies, gravity fill was the main mechanism, while suction fill (vacuum effect when lower punch is dragged downwards) is necessary for complete flow into smaller dies (25). The same sets of tooling were compared in a follow-up study, where three other parameters were investigated: angle of feeder paddles, feed wheel speed (5–17 rpm), and turret rotation (25–45 rpm) (26).

Apart from the discussed above in-depth studies on select phenomena, several papers report the application of different aspects of quality by design (QbD) approach to characterization and optimization of minitablets as a dosage form. However, to the authors’ knowledge, none of them comprehensively entails formulation and processing aspect in a practical industrial setting. For instance, Barmpalexis et al. employed advanced regression modeling and data analysis tools based on artificial intelligence along with design of experiments (DoE), but the studied input variables (filler and lubricant types, contents of different particle size fractions in the blend) were limited only to formulation aspects. Additionally, an eccentric tablet press was used, which lacks industrial relevance (17). Formulation-only DoE was also described in a case study using Box-Behnken design, where mannitol content in MCC (%) as filler combination, swelling pressure of a superdisintegrant, and surface area of Aerosil as a glidant were input variables and the responses were mass, thickness, content uniformity, and disintegration time of risperidone ODMTs. Based on several regression models including linear, quadratic, and interaction effects, orodispersible tablet compositions were optimized; however, no information is given on the impact of any compression process variables on ODMT quality attributes (27). Several aspects of QbD were included in the study by Iurian et al., such as Ishikawa diagram for identification of critical parameters and D-optimal design with the aim to construct the design space for 2-mm orodispersible minitablets. The investigated factors were lubricant type (sodium stearyl fumarate *vs.* magnesium stearate), lubricant concentration (1–4%), and compression load applied on a Gamlen tablet press (200–400 kg). The analyzed responses were ODMTs disintegration time and crushing strength, as well as the results of dynamic compaction analysis, such as work of compression, elastic recovery, and detachment or ejection stress. The resulting design space with sodium stearyl fumarate as the optimal lubricant with constraints related only to minitablets’ critical quality attributes was markedly broader than when additional constraints related to compaction stresses were incorporated. While the cited report presents insights into the effects of lubricant and compression load, the results cannot be directly referred to an industrial practice: only placebo blends were studied with sole focus on lubricants, while other excipients fell outside its scope. Moreover, the equipment used did not allow to consider processing parameters relevant in rotary tablet presses (20).

Considering all these aspects, it must be stated that to the authors’ knowledge in the publicly available literature, there is an absence of thorough studies which would simultaneously evaluate the effect of the majority of relevant formulation and compression process factors and their interactions on the critical quality attributes (CQAs) of orodispersible minitablets under QbD paradigm. Therefore, the following case study on melatonin ODMT optimization, employing the tools of DoE to construct a robust design space in an industrially relevant setting (semi-technical scale) aims to fill this gap. In this study, melatonin was used as the active substance. The development of ODMT formulation for this molecule seems to be crucial in the context of treating sleep disorders in pediatric patients, geriatric patients, and those with swallowing problems (28–30).

The objective of this work was to implement the DoE concept into the development phase of ODMTs. The content is organized into two parts. The first one is devoted to the identification of raw materials and process variable strength and its impact on the examined product quality attributes to identify those that are of the greatest importance. For this purpose, the methodology of Plackett-Burman screening design was applied. The second part of the paper is focused on the optimization of minitablet compression process. The influence of critical process parameters (CPPs) such as main compression force and tableting speed on tablet CQAs was investigated with the use of full factorial design and fractional factorial design. Finally, the mathematical models describing relationships between CPPs and CQAs served to establish the design space for the tableting process.

## MATERIALS AND METHODS

### Materials

Minitablet composition was based on two types of co-processed excipients (CPE): GalenIQ™ 721 (*further in text abbreviated to GIQ*) (Beneo GmbH, Germany) and Granfiller-D™ 215 (*further in text abbreviated to GFD*) (Daicel Corporation, Japan). Crospovidone (BASF) was selected as the disintegrant. Aspartam (Hyet Sweet S.A.S., France) was used as the sweetener agent and orange flavor type Q-128155 (Givaudan, Switzerland) was applied. Vegetable magnesium stearate (FACI, Italy) was used as a lubricant. Micronized and non-micronized (coarse grade) melatonin (Flamma S.p.A., Italy) was used as the active pharmaceutical ingredient (API). All samples were kindly provided by the manufacturers.

### Methods

#### Determination of Particle Size Distribution by Laser Diffraction Method

The volume median diameters d0.1 μm, d0.5 μm, d0.9 μm of the melatonin particles were determined by laser light diffraction method (Malvern Mastersizer 2000, Malvern Instruments). The methodology was the same as in our previous work (31). Briefly, the micronized melatonin powder samples were dispersed in n-heptane with the addition of 3–4 drops of surfactant. The suspension was stirred on a magnetic stirrer. Then, the sample was submerged in an ultrasonic bath. The coarse grade melatonin powder samples were dispersed in cyclohexane and stirred on a magnetic stirrer. The suspension was submerged in an ultrasonic bath. Particle sizes were measured in six replicates per batch using wet dispersion unit Hydro 2000S(A). In this study, median particle size d0.5 μm was selected as the most discriminative attribute (31).

#### Examination of Particles with a Scanning Electron Microscope (SEM)

Morphological structures of the active substance and main fillers, GIQ and GFD, were evaluated using a TM-1000 (Hitachi, Japan) compact electron microscope at a magnification of 250 and 1000×. For better image quality, the sample was initially dry sprayed with a gold layer (approx. 25 nm) in a K-550X (Quorum Technologies, U.K.).

#### Preparation of Powder Mixtures Used in Plackett-Burman Design Tests

Sixteen powder mixtures were prepared according to the composition presented in Table I. All the mixtures contained 8.35% w/w of API, 0.5% w/w of flavor, and 3.0% w/w of magnesium stearate. Since the application of GIQ required an additional disintegrating agent, 3.0% w/w of crospovidone was added. An amount of main filler (GFD or GIQ) was adjusted to reach 6.0 mg of the total nominal tablet mass. In all cases, the active ingredient was initially blended in a small metal vessel with part of the main filler, flavor, and crospovidone (in the case of mixtures based on GIQ) and sieved through a 0.71 mm sieve to improve the mixture uniformity. Then, premix was placed into the container of a TMG1/6(Glatt) laboratory high-shear mixer, sandwiched between two equal layers of the remaining main filler. Finally, the powder mixture was blended for 5 min. with impeller and chopper set at 300 rpm. Following the mixing, the container content was discharged into a metal vessel, and ten 100 mg samples of powder were collected from ten representative points for blend uniformity evaluation. In the next step, each powder blend was additionally blended with magnesium stearate. The premixing and sieving procedure was applied similarly as for the previous stage. Final blending was conducted using the same high-shear mixer. However, the impeller and chopper were only switched on for 1 min.

**Table I:** Composition of the Minitablets According to Plackett-Burman, Fractional Factorial, and Full Factorial Design

Ingredient name	Ingredient amount in the minitablet (mg)		
	Design type		
	Plackett-Burman^*1*^	Plackett-Burman^*2*^	Fractional factorial/fullfactorial
Melatonin	0.501	0.501	0.501
GalenIQ^TM^ 721	5.109	-	-
Granfiller-D^TM^ 215	-	5.289	5.347
Crospovidone	0.180	-	-
Flavor	0.030	0.030	0.008
Aspartame	-	-	0.024
Magnesium stearate	0.180	0.180	0.120

^*1*^Run No.: 2–3; 6–7; 10–11; 14–15

^*2*^Run No.: 1–4; 5–8; 9–12; 13–16

#### Preparation of Powder Mixture Used in Fractional and Full Factorial Design

To conduct full and fractional factorial design tests, powder mixture composition was prepared as presented in Table I. Based on the results of the previous experimental steps, only GFD was used as the main filler. The micronized melatonin remained in the amount of 8.35% w/w. Regarding flavor and magnesium stearate, the amount was decreased to 0.13% w/w and 2.0% w/w, respectively. Moreover, to improve the taste of the minitablets, sweetener (Aspartame) was applied in the amount of 0.4% w/w. Such a correction in the composition of minitablets was introduced, since after the Plackett-Burman experiments, further independent optimization work was carried out. It revealed that smaller amount of lubricant (2% instead of 3%) did not increase ejection force and it was beneficial for palatability attribute. In effect, the quantity of the main filler was very slightly increased from 88.15 to 89.12%. The total nominal minitablet mass remained equal to 6.0 mg. The procedure of the mixture preparation was adapted from the Plackett-Burman design stage.

#### Preparation of Minitablets

Minitablets were manufactured on an IMA Pressima AX rotary tablet press equipped with multi-tip punches (Natoli). Each punch was fitted with 5 or 13 tips. Single minitablet was round, biconvex, 2.0 mm in diameter, with 2.4 mm cup radius and mass of 6.0 mg. Minitablet samples (*n*=300) were taken periodically and their mass variation (SD) and physical attributes were analyzed.

#### Blend Uniformity

Each powder sample was dissolved in 40% methanol solution. API content was determined using a validated UHPLC method with UV-VIS detection at 222 nm. A detailed description of the sample preparation method and measurement conditions is given in our previous work (31).

#### Determination of Minitablet Resistance to Crushing, Disintegration Time, and Friability

The resistance to crushing (N) of minitablets was checked using a texture analyzer (Shimadzu Autograph AGS-X) equipped with TrapeziumX software. Each minitablet was placed in the platform’s center in an upright position and then exposed to the rod shape probe. During the test, the probe (6 mm diameter) moved downward at the constant speed of 2 mm/min. The applied force was measured by the sensor (of 10 N sensitivity) and recorded by the software. The resistance to crushing of minitablets was considered as the highest force value corresponding to the maximum of the first, significant peak (breakpoint) in the curve of force in the function of displacement. Ten randomly selected minitablets were tested from each batch and the results are expressed as mean values ± SD. Additionally, resistance to crushing was determined according to Ph. Eur. (2.9.8) using a hardness tester (Erweka TBH225D).

Determination of the disintegration time (s) was carried out using both a compendial (Ph.Eur. 2.9.1) method (Erweka ZT322) and according to different procedure with texture analyzer. Before the test, the minitablet was placed in a horizontal position on a Petri dish (located on the platform’s center), directly under the probe, and the measurement was initiated. The probe moved downward at the constant speed of 1 mm/min. When the force reached the value of 1 N, the probe stopped. At the same time, 0.5 ml of purified water was added using an automatic pipette so that the entire minitablet was covered with the medium. Break-up time was defined as the difference between the time when the force acting on the tablet reached 0 N (the tablet fully disintegrated) and the time when the force was 1 N (the time when water was added). Ten randomly selected minitablets from each batch were tested. The results are presented as mean values ± SD. Finally, the spread of resistance to crushing and disintegration time parameters was calculated as the difference between the maximal and minimal value. Friability was evaluated according to Ph. Eur. (2.9.7) using a friability tester (Erweka TAR120). In all measurements, 6.5 g sample of minitablets was tested.

#### Determination of Minitablet API Content Uniformity and In Vitro Dissolution

To determine API content uniformity for a sample of 10 units, a single tablet was dissolved in 40% methanol solution with sonication. API content was determined by means of a validated UHPLC (Waters/Agilent) method. The uniformity of dosage units was expressed by acceptable value (AV) < 15, according to Ph. Eur. (2.9.40). *In vitro* dissolution test was performed at sink conditions using a paddle apparatus (Ph. Eur. apparatus 2, Erweka at 37 ± 0.5°C, 50 rpm, 900 mL fill volume, sampling time points: 5, 10, 15, and 20 min.). A standard compendial dissolution medium 0.1 M HCl was used. A detailed description of the both methods is given in our previous work (31).

#### Statistical Analysis

All experimental designs and statistical evaluations were conducted with Statistica 13.3 (Tibco) software.

##### Plackett-Burman Design

The Plackett-Burman screening design was selected to identify the strength and the importance of compression step variables represented by process parameters and quality attributes of raw materials. This type of design identifies the main effects based on outcomes of the trials performed according to the matrix where low and high levels of independent variables are tested (Table II). The statistical tool enables us to identify factors that may be responsible for potentially the greatest variability in product quality attributes in the case of non-repeatability of the process parameters or the qualitative variability of raw materials.

**Table II:** Independent Variables Values Used in Plackett-Burman Design

Independent variables	Low level (−)	High level (+)
Amount of punches (pcs)	2	6
Table speed (rpm)	16	25
Feeder speed (rpm)	10	15
Pre-compression force (kN)	0.5	1.0
Main compression force (kN)	2.0	8.0
Carrier type	Granfiller-D^TM^ 215	GalenIQ^TM^ 721
Melatonin type	Micronized	Non-micronized

**Fig. 1:**
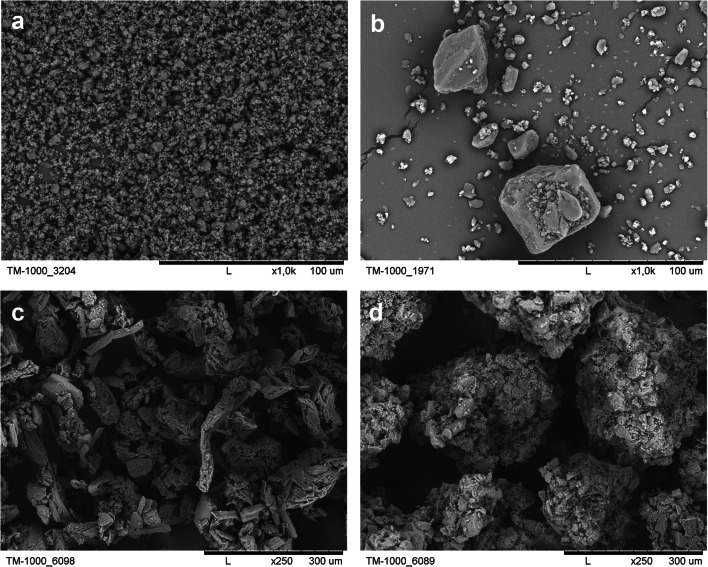
SEM images of micronized (**a**) and non-micronized (**b**) MEL (magnification 1000×) and two main fillers: GFD (**c**) and GIQ (**d**) (magnification 250×)

Based on previous knowledge and risk assessment, seven potentially critical variables were investigated: amount of punches, table speed, feeder speed, pre-compression force, main compression force, carrier type, and melatonin type. The potential influence of abovementioned variables was investigated on such output variables as follows: blend uniformity (expressed as RSD value), ODMT weight spread (difference between maximum and minimum values), API dissolution at 15′, resistance to crushing, resistance to crushing spread, friability, and content uniformity. The trials were carried out in randomized order using 5-tipped tools (Figure 1, Supplementary material).

##### Full Factorial Design

The 3^2^ full factorial design was created to determine the influence of the main compression force and tableting speed on such dependent variables as follows: resistance to crushing, resistance to crushing standard deviation (SD), disintegration time, tablet weight variation (SD), chosen based on the results of Plackett-Burman screening design. In the case of resistance to crushing and disintegration time, two types of responses were evaluated: the results obtained with compendial methods and with texture analyzer (marked as “TXT”). In the case of 3^2^ full factorial experimental design, input variable values are set on three levels, that is low, medium, and high, labeled as −1, 0, and +1, respectively. In total, twelve trials were performed, including 3 replications of the center point (10C, 11C, and 12C) (Table III). All parameters other than the tested factors were kept constant. This type of matrix enables to investigate quadratic relationships and interactions existing between independent variables and responses, allowing thorough characterization of potential non-linear relationships in the process. Simultaneously, it saves time and experimental resources.

**Table III:** 3-Level Full Factorial Experimental Design Scheme Applied in the Investigation of the Influence of Main Compression Force and Tableting Speed on Tablet CQAs

Run number	Independent variable	
	Main compression force (kN)	Table speed (rpm)
1	−1	−1
2	−1	0
3	−1	+1
4	0	−1
5	0	0
6	0	+1
7	+1	−1
8	+1	0
9	+1	+1
10C	0	0
11C	0	0
12C	0	0

All polynomial coefficients were estimated using the least-squares method. The statistically significant terms were identified by the analysis of variance (ANOVA) at *p*<0.05. Model building was performed by backward elimination of statistically insignificant terms to give the most simplified form of the predictive equations describing the relationships between input and output variables. The Shapiro-Wilk statistic (*α*=0.05) was applied to confirm whether the residuals have normal distribution (Figure 7^3^, Supplementary material). The adjusted coefficient (*R*^2^_adj_) values were designated to determine to what extent the developed model explains the variability of the dependent variables. Lack-of-fit statistic was tested for all models to confirm its non-significance, indicating model validity. (Table I, Table II Supplementary material).

##### Fractional Factorial Design

>The 3^(3-1)^ fractional factorial design was applied to determine the influence of the main compression force, tableting speed, and also the amount of 13-tipped punches on dependent variables: resistance to crushing, resistance to crushing SD, disintegration time, friability, and resistance to crushing TXT (as determined by texture analysis). In the case of a larger number of variables on multiple levels, the fractional designs are the better choice. In practice, the use of complete plans means the need of performing a very large number of tests in order to examine all variants. The use of fractional designs allows for deduction based on a smaller number of conducted experiments (Table IV). Despite the sacrifice of interaction effects, the main ones can still be correctly estimated. The experimental matrix also included 3 replicates of central points to calculate pure error.

**Table IV:** 3^(3-1)^ Fractional Factorial Experimental Design Scheme Applied in the Investigation of the Influence of Main Compression Force, Tableting Speed, and Amount of Punches on Tablet CQAs

Run number	Independent variable		
	Main compression force (kN)	Table speed (rpm)	Amount of punches (pcs)
1	−1	−1	−1
2	−1	0	1
3	−1	1	0
4	0	−1	1
5	0	0	0
6	0	1	−1
7	1	−1	0
8	1	0	−1
9	1	1	1
10C	0	0	0
11C	0	0	0
12C	0	0	0

## RESULTS AND DISCUSSION

### Raw Material Properties

In order to design an ultralow dose of melatonin (< 0.5 mg), ODMT formulation with statistical tools, selection of the excipient should be initially carried out. In the preformulation study (31), it was indicated that Granfiller-D^TM^ 215 (GFD) would be the best candidate for the final formulation. In contrast, GalenIQ^TM^ 721, which was finally rejected, showed many interesting properties like excellent flowability and beneficial palatability aspects. Both excipients present diverse morphological properties (especially in solidity and circularity parameters). For this reason, it was decided to use both main fillers to evaluate statistical impact on product critical quality attributes (CQAs). Granfiller-D^TM^ 215 is a typical co-processed excipient, a combination of D-mannitol, microcrystalline cellulose, carmellose, and crospovidone. All three components support D-mannitol in acceleration of tablet disintegration process (carmellose works as a wicking agent, crospovidone works as a swelling agent, whereas cellulose creates an insoluble matrix body). GalenIQ^TM^ 721 (isomalt) is a combination of 6-O-α-D-glucopyranosyl-D-sorbitol (1,6-GPS) and 1-O-α-D-glucopyranosyl-D-mannitol dihydrate (1,1-GPM). Although it is not a co-processed excipient, according to declaration of manufacturer, GIQ is designed for ODT formulations. It should be noticed that its mechanism of action presents slow dissolution; therefore, an application of isomalt requires an additional disintegrating agent. Both reflect two different approaches to the formulation development. Thus, in this work, main fillers were used in combination with micronized and non-micronized melatonin to conduct a series of formulation and processing experiments with DoE methodology. Since particle size was indicated as the critical material attribute (CMA), the PSD examination was performed with laser diffraction method. The data for each component is presented in Table V. The results show that non-micronized melatonin is a heterogeneous material (the value of the span parameter is the highest in this group) and the median of particle size d0.5 is approx. 30 microns. In opposite, the micronized grade of API is much more uniform and the particle size is below 6 microns. In the case of carriers, the median of particle size is similar; however, the span value of GIQ is lower than GFD. The structure of all materials was also investigated with a scanning electron microscope. The SEM images are depicted in Figure 1. As can be seen, the structure of GIQ particles is spherical, with smooth and regular surface. In contrast, GFD particles have irregular, rather elongated shape with higher surface area.

**Table V:** Particle Size Distribution and span Values of Melatonin and Carriers Determined with Laser Diffraction Method, Mean ± Standard Deviation, *n*=6

Material	d0.1 μm	d0.5 μm	d0.9 μm	span
Micronized melatonin	0.12 ± 0.01	2.11 ± 0.27	5.75 ± 0.24	2.70 ± 0.28
Non-micronized melatonin	4.73 ± 0.24	30.86 ± 4.64	142.74 ± 17.78	4.55 ± 0.87
GalenIQ^TM^ 721	14.11 ± 0.53	61.90 ± 1.53	183.54 ± 9.42	2.74 ± 0.08
Granfiller-D^TM^ 215	10.65 ± 0.16	55.79 ± 0.22	190.74 ± 1.35	3.23 ± 0.02

It may be also assumed that such a difference in morphology will have an impact on powder flowability and ability to create homogenous mixtures with active ingredient. Therefore, flow properties were also checked (Table VI).

**Table VI:** Comparison of Main Fillers’ Flowability Properties

Main filler name	Flow angle (°)	Flowability (s/100g)	Bulk density (g/cm^3^)	Carr Index	Hausner ratio
GalenIQ^TM^ 721	80.1	17.5	0.41	14.58	1.17
Granfiller-D^TM^ 215	69.5	36.8	0.32	20.00	1.25

The results proved that the flowability of GIQ is much better than GFD. The Carr Index (CI) and Hausner ratio (HR) indicators (according to Ph.Eur. 2.9.36) also confirm such observation. Both classify GIQ as a good flowing powder (CI: 11–15%, HR: 1.12–1.18), whereas GFD as fair flowing powder (CI: 16–20%, HR: 1.19–1.25). It should be noticed that the difference in flowability parameter (s/100g) between GIQ and GFD is more than twofold, which means in practice significant contrast.

### Plackett-Burman Design

The Plackett-Burman screening design was applied to identify the process parameters and raw material attributes that may cause the greatest product CQAs change. This should enable better understanding of process variability and ensure higher level of its repeatability and predictability in the future. The values of input and obtained output variables are presented in Table VII.

**Table VII:** The Independent Variables and Responses Summary Obtained from Compression Experiments Using 5-Tip Tools, Performed According to Placket-Burman Design

Independent variables								Dependent variables						
Run no.	Amount of punches	Table speed (rpm)	Feeder speed (rpm)	Pre-compression force (kN)	Main compression force (kN)	Carrier type	API type	Blend uniformity RSD (%)	Tablet weight spread (mg)	API dissolution (%) after 15 min.	Resistance to crushing (N) (mean ± SD)	Resistance to crushing spread (N)	Friability (%)	Content uniformity (AV)
1	2	16	10	1.0	8	GFD	Micronized	0.60	1.5	96.0	11.30 ± 0.75	4	0.52	2.7
2	2	16	15	1.0	2	GIQ	Coarse	5.41	1.0	35.8	12.05 ± 0.80	11	0.30	18.1
3	2	25	10	0.5	8	GIQ	Coarse	13.46	1.0	34.6	16.65 ± 0.98	11	0.49	19.5
4	2	25	15	0.5	2	GFD	Micronized	1.04	0.6	95.3	5.37 ± 0.62	3	0.61	2.5
5	6	16	10	0.5	2	GFD	Coarse	4.56	1.0	93.5	4.45 ± 0.51	3	0.68	21.9
6	6	16	15	0.5	8	GIQ	Micronized	1.02	0.9	41.9	19.95 ± 3.05	16	0.37	6.2
7	6	25	10	1.0	2	GIQ	Micronized	1.08	0.9	42.4	10.40 ± 1.18	12	0.19	21.4
8	6	25	15	1.0	8	GFD	Coarse	5.56	1.4	87.0	9.00 ± 0.33	3	0.49	16.9

**Fig. 2:**
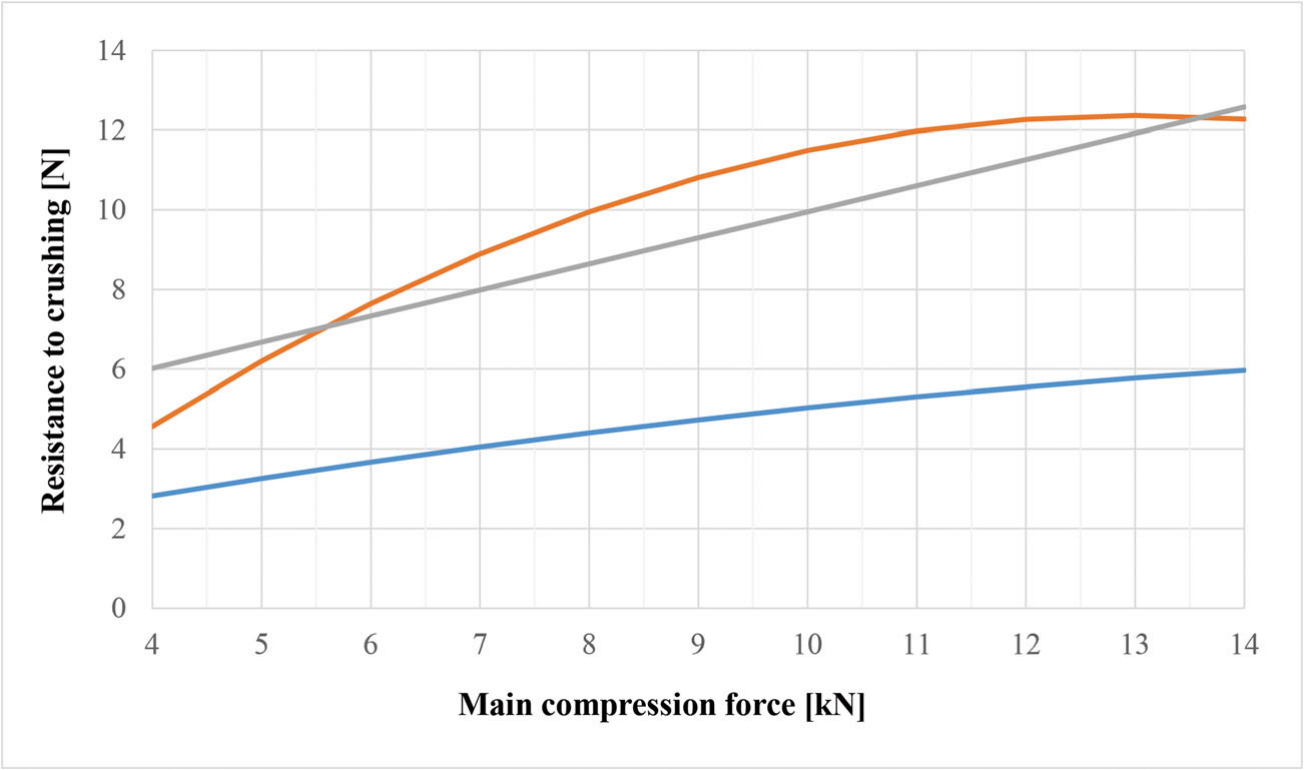
Effect of main compression force on tablet’ resistance to crushing parameter. Predicted values calculated based on the developed models. Blue line represents model based on pharmacopeia method, orange (full factorial design) and grey (fractional design) lines represent models based on data from texture analysis

Only two variables among the investigated ones may have a potential impact on the powder mixture homogeneity, namely the melatonin and main carrier type. Other variables of the tableting process should be treated as dim variables in this particular case. Thus, the obtained effect values should be treated as background noise, having no effect. It is demonstrated that the blend uniformity depends mainly on the melatonin type (its d0.5 value) that is used to prepare the powder mixture (effect 6.31; *p*-value <0.05) (Figure 2A, Supplementary material). The positive value of the effect indicates that with the switch of the variable from a lower to a higher level, and in practice, when changing the substance from micronized to non-micronized type, the value of the dependent variable which is blend uniformity expressed as RSD will increase. Increasing the value of the latter means in practice a decrease in homogeneity of the powder mixture. In other words, application of micronized API improves the homogeneity of the powder blend. Thus, micronized melatonin was finally selected for further investigation.

In the case of dissolution at 15 min. as response variable, the main carrier type (effect −54.27; *p*-value <0.05) and melatonin type (effect −6.18; *p*-value <0.05) played the major role (Figure 2C, Supplementary material). The use of GIQ as the main filler and non-micronized melatonin resulted in lower release at 15 min. from the dosage form (Table VII, Figure 5, Supplementary material). The use of GFD resulted in significantly faster release at 15 min. in comparison to GIQ regardless of API crystal size. The lowest result for GFD was 87%, whereas the highest result for GIQ was 42.4%. This strong impact is correlated with the different morphological structure of the main fillers and their chemical composition, i.e., the combination of mannitol, carmellose, and crospovidone in the co-processed GFD. It was no surprise that micronized API additionally supports faster dissolution of melatonin from the minitablets, since the smaller crystals size (d0.5 = 2.11 μm) results in expanded surface area, and finally enables faster dissolving. Uniformity of dosage units (expressed by AV value) was mainly affected by melatonin type (effect 10.90; *p*-value <0.05) (Figure 2G, Supplementary material). The use of micronized API resulted in a lower AV value. It might be explained by its ability to create more homogenous and stable mixtures: thanks to the adhesion to the porous surface of the filler (carrier) particles. The smaller API crystal size, the stronger the bonds between both components. Moreover, this finding confirms that the content uniformity of minitablets is sensitive to API particle size. Especially for low-potency minitablets, micronized APIs are beneficial, as inclusion or exclusion of a single large particle during die filling may have high impact on API absolute content and its uniformity (4). Such an interesting effect might be also explained by the morphology of the GIQ particles, which are characterized by a very smooth and regular surface (having lower surface energy). Thus, it is less prone to sticking of the small API particles on its surface. This is another factor in favor of using a micronized active in further development.

In the case of tablet weight spread and friability variables, no influence of the examined factors was found (Figure 2B, 2F, Supplementary material). On the basis of initial experiments and Plackett-Burman screening test results, a formulation prototype was defined. As the main filler, the GFD was selected and the micronized melatonin as the active ingredient.

### Full Factorial Design

In the next step, the influence of pre-selected process variables on product quality attributes was investigated. A three level, full factorial design was employed to establish the impact of two critical process parameters (CPPs), i.e., the main compression force (kN) and press table speed (rpm) on the values of dependent variables. The input values and response results are presented in Table VIII.

**Table VIII:** The Independent Variables and Response Summary Obtained by Applying Full Factorial Design

Trial number	Independent variable (CPP)		Dependent variables (CQA)					
	Main compression force (kN)	Table speed (rpm)	Resistance to crushing (N)	Resistance to crushing SD^*1*^	Disintegration time (s)	Weight SD^*1*^	Resistance to crushing TXT^*3*^ (N)	Disintegration time TXT^*3*^ (s)
1	6.0	13	3.60	0.96	3.00	0.25	8.102	6.821
2	6.0	18	3.80	1.38	3.00	0.19	7.509	6.185
3	6.0	23	3.60	0.78	3.00	0.13	7.317	6.378
4	10.0	13	4.80	1.34	2.00	0.28	10.977	11.349
5	10.0	18	5.10	1.28	4.00	0.24	10.937	12.364
6	10.0	23	4.70	1.24	2.00	0.31	10.896	12.331
7	14.0	13	5.70	1.05	5.00	0.30	11.893	15.087
8	14.0	18	6.10	1.44	4.00	0.34	12.104	16.840
9	14.0	23	6.10	1.05	3.33	0.36	12.811	16.398
10C^*2*^	10.0	18	5.20	1.49	4.67	0.09	12.291	11.992
11C^*2*^	10.0	18	5.10	1.51	5.50	0.11	11.453	11.992
12C^*2*^	10.0	18	5.30	1.18	2.67	0.09	12.316	11.157

^*1*^*SD* standard deviation; ^*2*^*C* center point; ^*3*^*TXT* texture analysis

The developed mathematical models that show the influence of CPPs on resistance to crushing parameter are presented below. The former was developed on the basis of data obtained with measurements by compendial method (Eq.1), dominant in the pharmaceutical industry. The latter was created based on data obtained with texture analysis (Eq.2).

1 $$Resistance\ to\ crushing=-0.014\ast {A}^2+0.558\ast A+0.804\kern4em \left(R^2_{\text{adj}}=0.94\right)$$

*A*—main compression force (kN); resistance to crushing is expressed in newtons (N)

2 $$Resistance\ to\ crushing\ TXT=-0.096\ast {A}^2+2.497\ast A-3.885\kern1.75em \left(R^2_{\text{adj}}=0.90\right)$$

*A*—main compression force (kN); resistance to crushing TXT is expressed in newtons (N)

The main factor that influences the tablets’ resistance to crushing is the value of main compression force. The effect and relationship are non-linear(Figure 2, orange line). Not surprisingly, the minitablet crushing strength increases with increasing compression force due to stronger binding of the material (1, 20). Interestingly, at higher compression force values, the response reaches a plateau, which suggests achieving maximum degree of plastic deformation and binding sites by the filler without fragmentation or brittleness which would compromise the ODMT’s mechanical stability. To the authors’ knowledge, such phenomenon has not been described for minitablet case studies, where usually linear increase in tensile strength was observed with increasing compression pressure (1, 18–20). This is likely explained by the differences in the used filler, as the cited works do not report the use of co-processed excipient GFD in this context.

Although both methods used to determine resistance to crushing indicate different values, they are correlated with each other (*R*=0.97). Both compendial method and texture analysis made it possible to develop similar mathematical models. However, the method of resistance to crushing measurement using texture analysis seems to be more recommended for the analysis of ODMTs. The reason is the low hardness values of the analyzed ODMTs when compared to conventional or non-orodispersible minitablets and higher accuracy of the method. While difficulties in applying traditional hardness testers for ODMTs are recognized in the literature and texture analysis has been employed to determine crushing strength in several studies (1, 19, 22, 27), to the authors’ knowledge up to date, no report has compared or modeled the results from the application of two testing methods. As demonstrated, both are correlated with each other and can be used for process characterization of ODMT compression, although texture analysis can be considered superior.

The model developed for disintegration time based on texture analysis is given by the equation (Eq. 3):

3 $$Disintegration\ time\ TXT=1.206\ast A-0.484\kern1.5em \left(R^2_\text{adj}=0.97\right)$$

*A*—main compression force (kN); disintegration time TXT is expressed in seconds (s).

The main factor responsible for the tablets’ disintegration time is the value of compression force used during the compaction process. With an increase in compression force, it was observed that the disintegration time also increased in a linear manner, which is obviously expected (1, 22, 27). Considering this together with resistance to crushing values, it must be stated that despite decreasing ODMT porosity as evident from longer disintegration, the mechanical properties of minitablets developed with GFD as the filler/binder are not affected. Unfortunately, for the data obtained by the compendial method, it was not possible to identify the factors influencing the disintegration time in a statistically significant manner. Consequently, we were unable to create a model. For tablet weight SD parameter, it was not found that any of the analyzed factors had a statistically significant influence on this CQA, which confirms uniform, repeatable filling of 2-mm dies with blend containing micronized API based on GFD excipient. Thus, it was not possible to develop a model. In the case of resistance to crushing SD parameter, no model was achieved that could explain the CQA variability to a satisfactory extent (*R*^2^_adj_ >0.5).

The developed models for resistance to crushing and disintegration are characterized by high *R*^2^_adj_, which indicates that over 90% of variability in the response value can be accounted for by the factor incorporated in the model, i.e., the effects of main compression force. Moreover, such models are expected to yield reliable predictions of the output values. To verify this, batches of 250,000 minitablets were compressed at 13.6 and 13.2 kN and the experimentally determined values of their resistance to crushing and disintegration time were compared with theoretical values calculated with model equations. Prediction error (%) was determined as the ratio of difference between the mean observed and predicted value to the observed one.

As can be seen in Table IX, the prediction error values are reasonable. The best agreement between theoretical values and observed ones occurred for the resistance to crushing results determined with texture analysis, which further confirms the superiority of this method. For the values measured with typical tablet hardness tester, the results are underestimated to a higher extent, which might be explained by high RSD values. High variability in minitablet tensile strength has been observed especially with decreasing die sizes. However, the presented comparison here strongly suggests that in the case of our tablets, high RSD of resistance to crushing determined with compendial method is due to unsuitable instrumental accuracy and precision, and not due to processing faults.

**Table IX:** Verification of Predictions Developed with Full Factorial Design Models

Batch number	Main compression force (kN)	Resistance to crushing			Resistance to crushing TXT			Disintegration time TXT		
		Predicted (N)	Observed (N)	Error (%)	Predicted (N)	Observed (N)	Error (%)	Predicted (s)	Observed (s)	Error (%)
A	13.6	5.8	6.5 (*n* = 30)	10.8	12.3	12.6 (*n*=10)	2.4	15.9	14.8 (*n*= 10)	-7.4
B	13.2	5.7	6.3 (*n*=30)	9.5	12.4	12.9 (*n*=10)	3.9	15.4	13.9 (*n*= 10)	-10.8

### Fractional Factorial Design

A 3^(3-1)^ fractional factorial design was employed to establish the influence of three critical process parameters (CPPs): main compression force, tableting speed, and amount of multi-tip punches on the values of dependent variables. The input values and response results are presented in Table X.

**Table X:** The Independent Variables and Responses Summary Obtained from Fractional Factorial Design

Trial number	Independent variable			Dependent variables					
	Main compression force (kN)	Table speed (rpm)	Amount of punches (pcs)	Resistance to crushing (N)	Resistance to crushing SD^*1*^	Disintegration time (s)	Friability (%)	Weight SD	Resistance to crushing TXT (N)
1	6	13	2	4.00	1.02	5.00	0.44	0.16	7.52
2	6	18	10	4.10	1.01	4.00	0.44	0.22	6.27
3	6	23	6	4.20	1.24	2.33	0.43	0.19	6.70
4	10	18	6	4.30	0.83	3.67	0.33	0.09	10.29
5	10	18	10	5.20	1.15	4.00	0.37	0.28	11.04
6	10	23	2	4.70	0.94	3.67	0.43	0.14	7.17
7	14	13	6	7.60	1.28	5.00	0.36	0.43	12.72
8	14	18	2	5.40	0.77	2.00	0.42	0.18	11.81
9	14	23	10	5.50	1.55	3.67	0.41	0.32	11.69
10C^*2*^	10	18	6	4.70	0.87	4.33	0.33	0.09	11.10
11C^*2*^	10	18	6	4.40	1.43	5.33	0.33	0.11	11.63
12C^*2*^	10	18	6	6.30	1.34	4.33	0.45	0.36	11.49

^*1*^*SD*, standard deviation; ^*2*^*C*, center point

The model developed for resistance to crushing parameter based on texture analysis is given by the equation (Eq. 4):

4 $$Resistance\ to\ crushing\ TXT=0.655\ast A-0.190\ast B+6.824\kern2.75em \left(R^2_\text{adj}=0.73\right)$$

*A*—main compression force (kN);*B*—table speed (rpm); resistance to crushing TXT is expressed in newtons (N).

The factors that influence the tablets’ resistance to crushing TXT are main compression force and tableting speed. As the compression force increases, a linear increase in the hardness of the tablets is observed. This is indicated by the positive value of the effect (5.24; *p*-value <0.05). Increasing the table speed causes a linear drop in the resistance to crushing of the obtained tablets. It is assigned to the negative sign of the effect (−1.90; *p*-value <0.05). This phenomenon may be caused by shorter dwell time with the increasing turret rotation. The shorter time the powder spent under pressure inside the die, the fewer permanent bonds between the compressed powder particles were produced. The absolute value of the tableting speed effect is smaller if we compare it with the strength of the compression force effect. In the publicly available literature on minitablets, the influence of turret speed on tablet tensile strength has not been explored extensively. In the study by Goh et al., this factor was not identified as statistically significant, which was tentatively attributed to the particular formulation’s insensitivity to narrowing compression profile, increased strain rate, and shorter dwell times with increasing turret speed (26).

The *R*^2^_adj_ value of the achieved model is lower than in case of the first one built based on data from full factorial design. It means that less variability in tablet resistance to crushing is explained by this model. The difference most likely is related to different datasets being studied and reduced number of points, where relationships are not explored over the whole experimental space and effects cannot be estimated as reliably as with full factorial design. Nevertheless, similar dependence of resistance to crushing on compression force was detected with the same effect sign, and the influence of tableting speed can be considered as of minor importance. Moreover, the results of fractional factorial design experiments confirmed that the number of punches applied in the rotary tablet press was not a significant factor for any of the responses. However, no comparison with literature findings can be made; as to the authors’ knowledge, no study on minitablets has considered this as a variable for investigation.

For dependent variables, resistance to crushing, resistance to crushing SD, disintegration time, weight variation, and friability, no models were developed due to lack of statistically significant impact of independent variables on quality attributes or because simple achieved models did not explain the variability of CQAs to a satisfactory extent (*R*^2^_adj_ > 0.5).

### Design Space

The design space (DS) was created in order to define the combination of input variables, the use of which in the production process guarantees obtaining a product with the desired quality characteristics. The two CQAs, namely resistance to crushing TXT and disintegration time TXT, were chosen to establish the design space. The DS was graphically represented by means of 2D graphs based on the developed equations. The following constraints were taken into consideration:

- The average value of resistance to crushing TXT should be more than 9.5 N. From our experience, it is not possible to achieve tablets harder than 14 N. Above this border plateau is observed. In order to set a limit that would ensure that all tablets would meet the requirements, taking into account their natural variance, it was assumed that the standard deviation for this parameter is 0.5 N. Thus, the value of resistance to crushing TXT of single ODMT should be not less than 8.0 N. This constraint is based on the fact that values below 8.0 N were related to unacceptably high friability (> 0.5%).
- The average disintegration time TXT should be less than 30 s, typically for ODMTs.

**Fig. 3:**
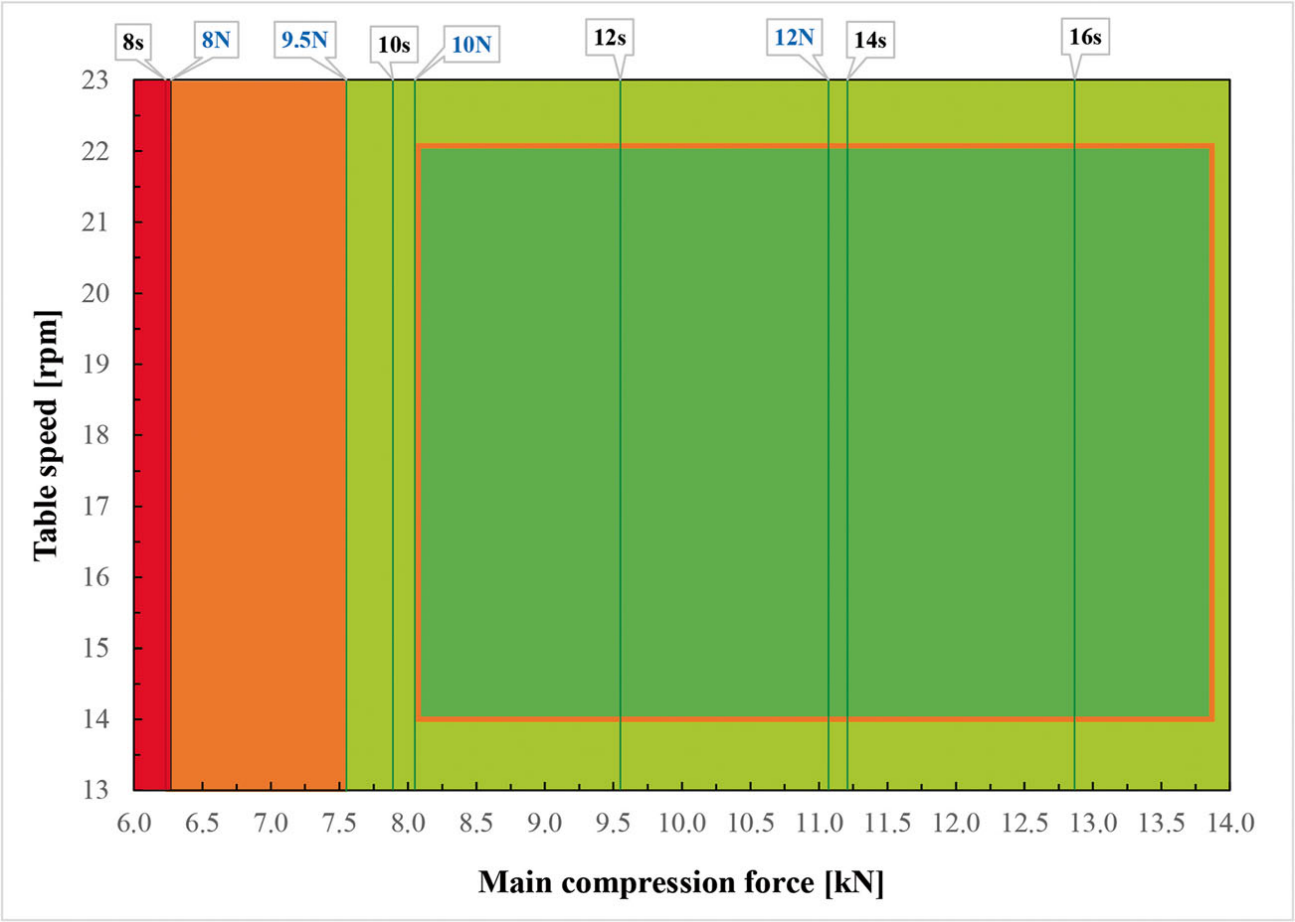
Compression design space with PAR limits established based on full factorial design. Green area shows the values of both factors that enable to produce tablets with all quality attributes within the specified limits. Red area represents the values of factors whose application will result in achieving a product with at least one of the CQAs out of the limits. Orange area shows value of resistance to crushing TXT acceptable for single tablets but not acceptable as an average value. Red square and the area closed inside represents PAR limits. The top horizontal axis shows the values of resistance to crushing (blue) and disintegration time (black)

**Fig. 4:**
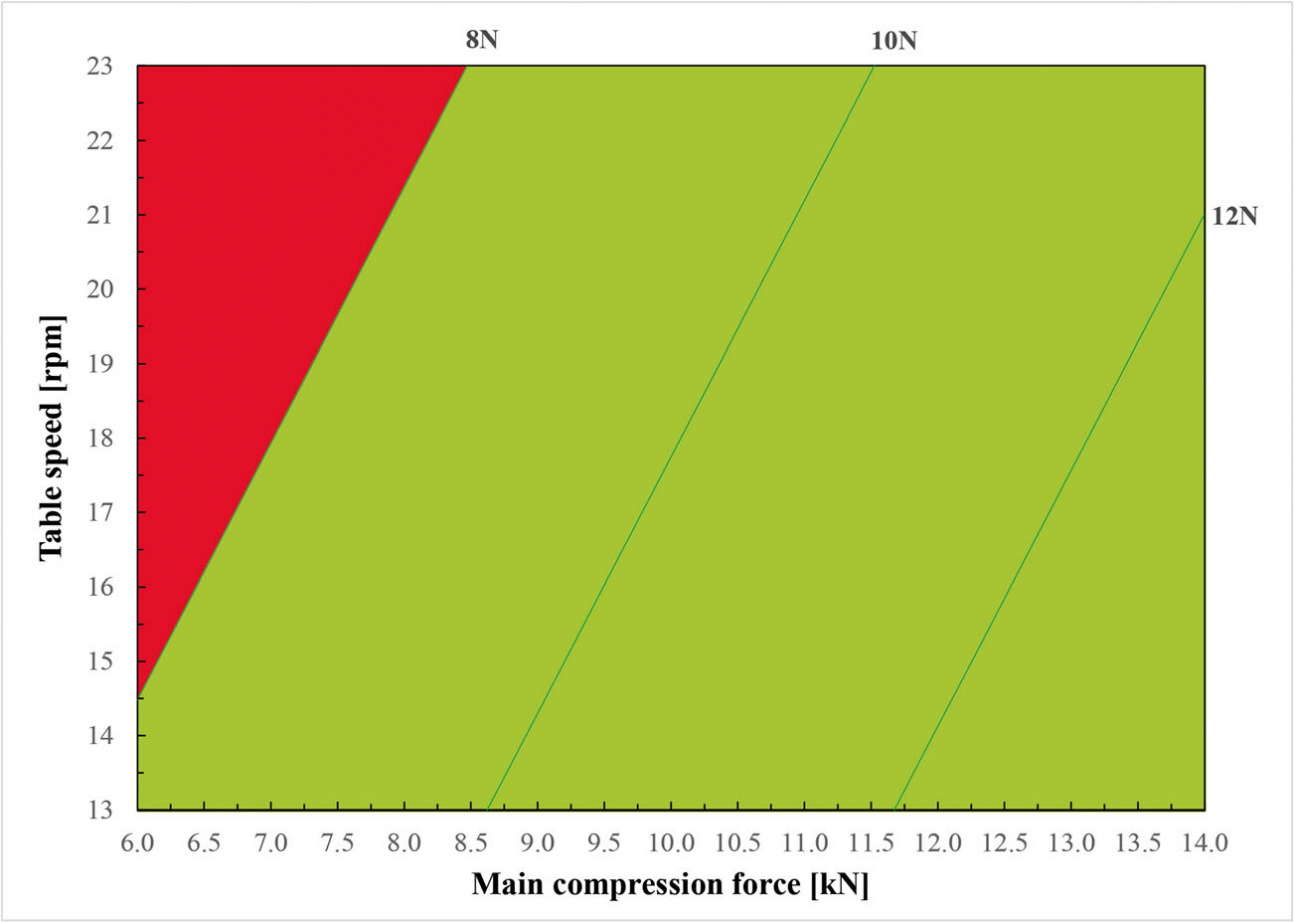
Compression design space established based on fractional factorial design. Green area shows the values of both factors that enable to produce tablets with all quality attributes within the specified limits. Red area represents the values of factors whose application will result in achieving a product with at least one of the CQAs out of the limits

Within tested ranges of CPPs, average resistance to crushing TXT exceeds the lower range only if tableting is performed using main compression force lower than 7.55 kN. The requirement that disintegration time TXT should be less than 30 s is fulfilled in the whole range of settings. Thus, by overlapping the requirements for both CPPs, the design space was established. It shows the ODMTs of required quality can be manufactured by using the main compression force higher than 7.55 kN (green area on Figure 3). In conclusion, the created design space enabled the compression process optimization and served to indicate the Proven Acceptable Range of compression force. It will find application during the routine production. Additionally, another DS was alternatively simulated based on the models developed with the use of fractional factorial design (Figure 4), where acceptable lower limit of compression force is marginally dependent on tableting speed. This illustrates the difference in the datasets and statistical treatment used for building model equations according to various matrix designs and the inclusion or exclusion of independent variables of borderline significance, i.e., table speed. Nevertheless, the CPPs ranges established based on the assumptions of both full and fractional factorial design models are similar and mostly overlapping.

## CONCLUSION

This paper presented a successful optimization of formulation and compression process of orodispersible minitablets containing melatonin under quality by design approach, with the application of design of experiments. Placket-Burmann screening design was used to identify the strongest factors affecting the product's critical quality attributes. The carrier type and particle size of API were found to exert the strongest influence, which prompted the choice of micronized melatonin and Granfiller-D^TM^ 215 for the optimized formulation. Full and fractional factorial designs were employed to develop models describing the relationships between tableting process parameters and the ODMT resistance to crushing and disintegration time. Moreover, compendial methods for the determination of these attributes were compared with texture analysis, demonstrating better suitability of this analytical tool for the evaluation of orodispersible minitablets. The developed model equations displayed good agreement between the calculated and observed values (as evident from *R*^2^_adj_ = 0.90–0.97) and the predictive power was successfully verified on additional datasets. As a result, the design space for the compression process of melatonin ODMTs was established and Proven Acceptable Range for the tableting operation was confirmed.

Therefore, the presented case study demonstrates an example of systematic and successful optimization of melatonin ODMTs in an industrially relevant setting. As indicated by the processing ranges of the design space, the chosen formulation was flexible and robust towards tableting speed and compression force values. Based on these results, tableting parameters with appropriate safety margin were set as Proven Acceptable Range, ensuring repeatable and robust routine manufacturing owing to the application of quality by design principles in the development phase.

## Supplementary Information

ESM 1 (DOCX 2057 kb)
