# Supplementary material for: Implementing the Design of Experiments (DoE) Concept into the Development Phase of Orodispersible Minitablets (ODMTs) Containing Melatonin
Source: AAPS PharmSciTech. 2022 Jan 20;23(1):60. doi: 10.1208/s12249-021-02185-6 (PMC8816488; doi:10.1208/s12249-021-02185-6)
Supplement: Supplementary file 1 — (DOCX 2057 kb) [file 12249_2021_2185_MOESM1_ESM.docx]

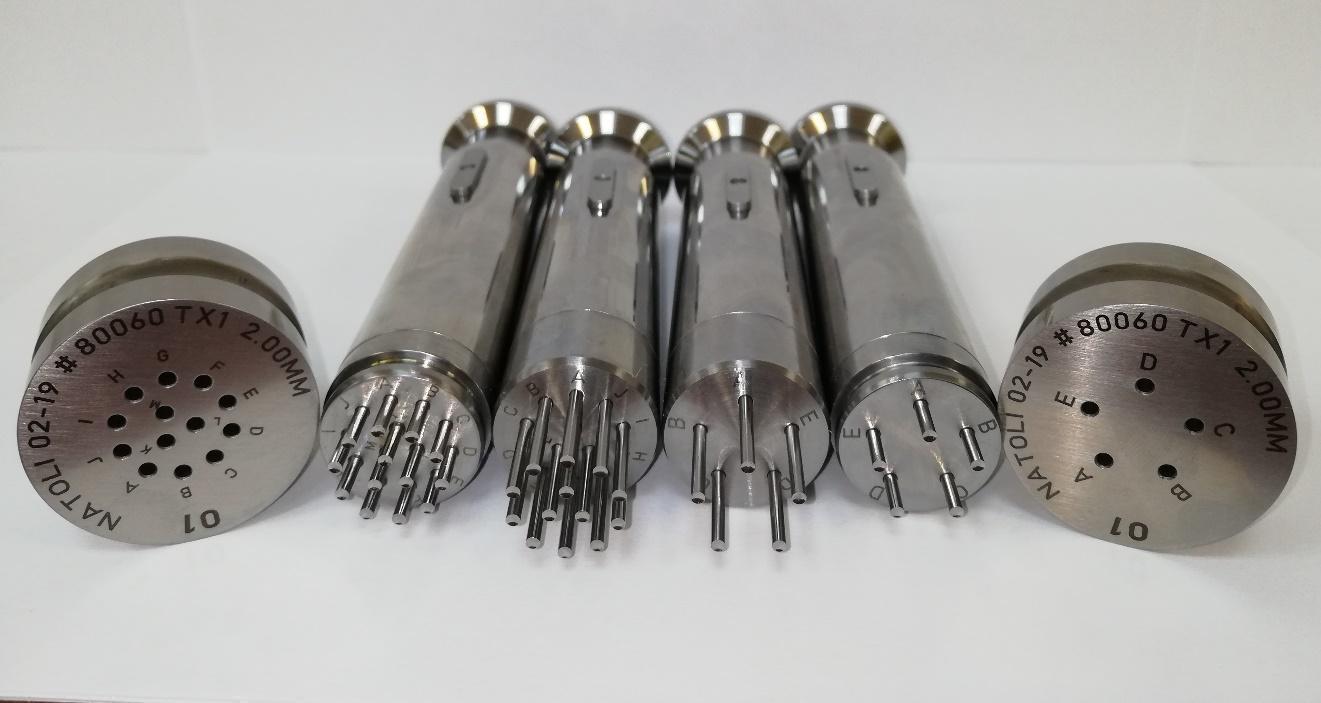


**Figure 1.** Multi-tip tools. 5-tipped punches and die (right) used in Placket-Burman screening design and 13-tipped punches and die (left) used to establish Design Space applying full factorial and fractional factorial design.

**Figure 2**. Placket Burman – Pareto charts.

A – Blend Uniformity

**
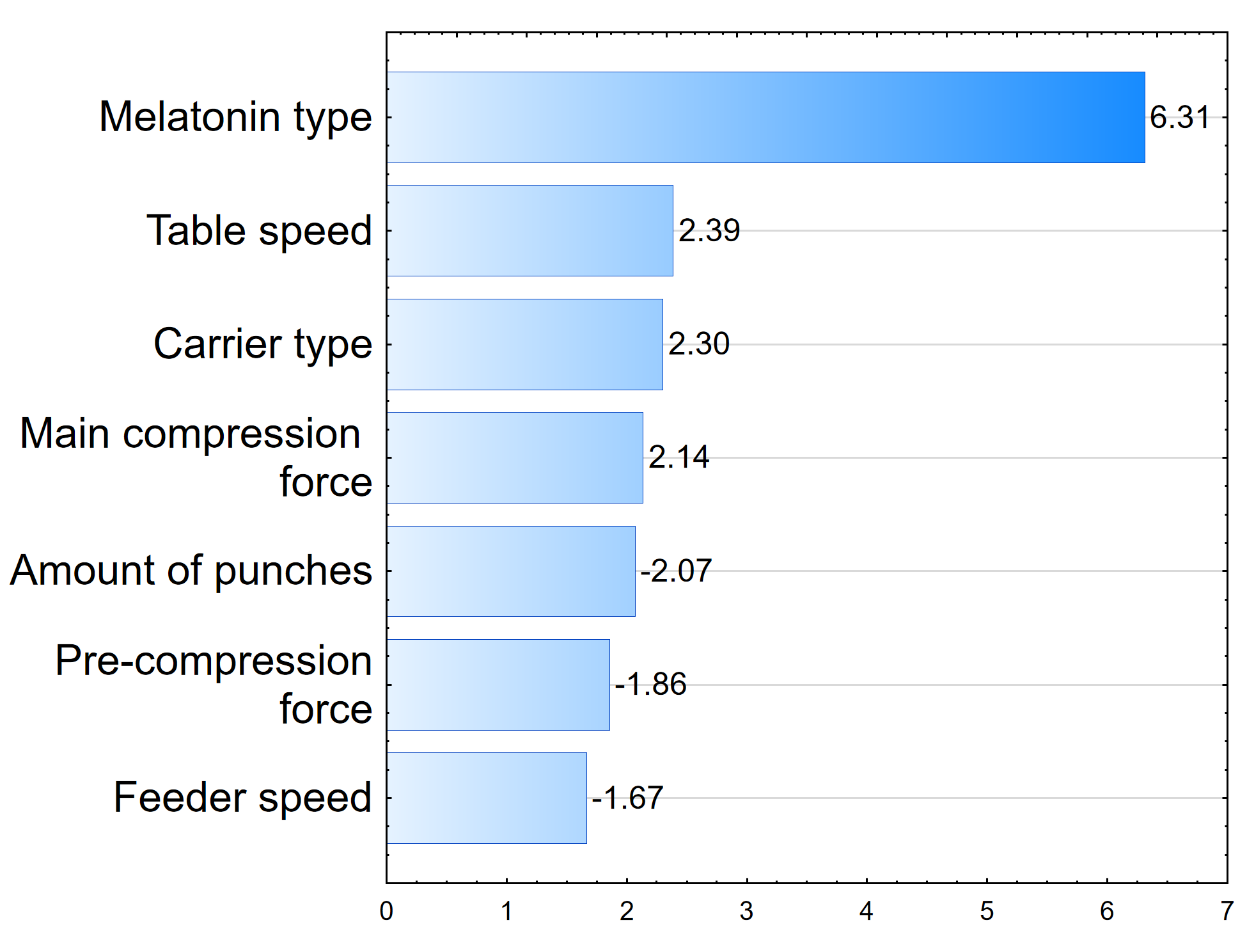
**

B – Tablets weight spread

**
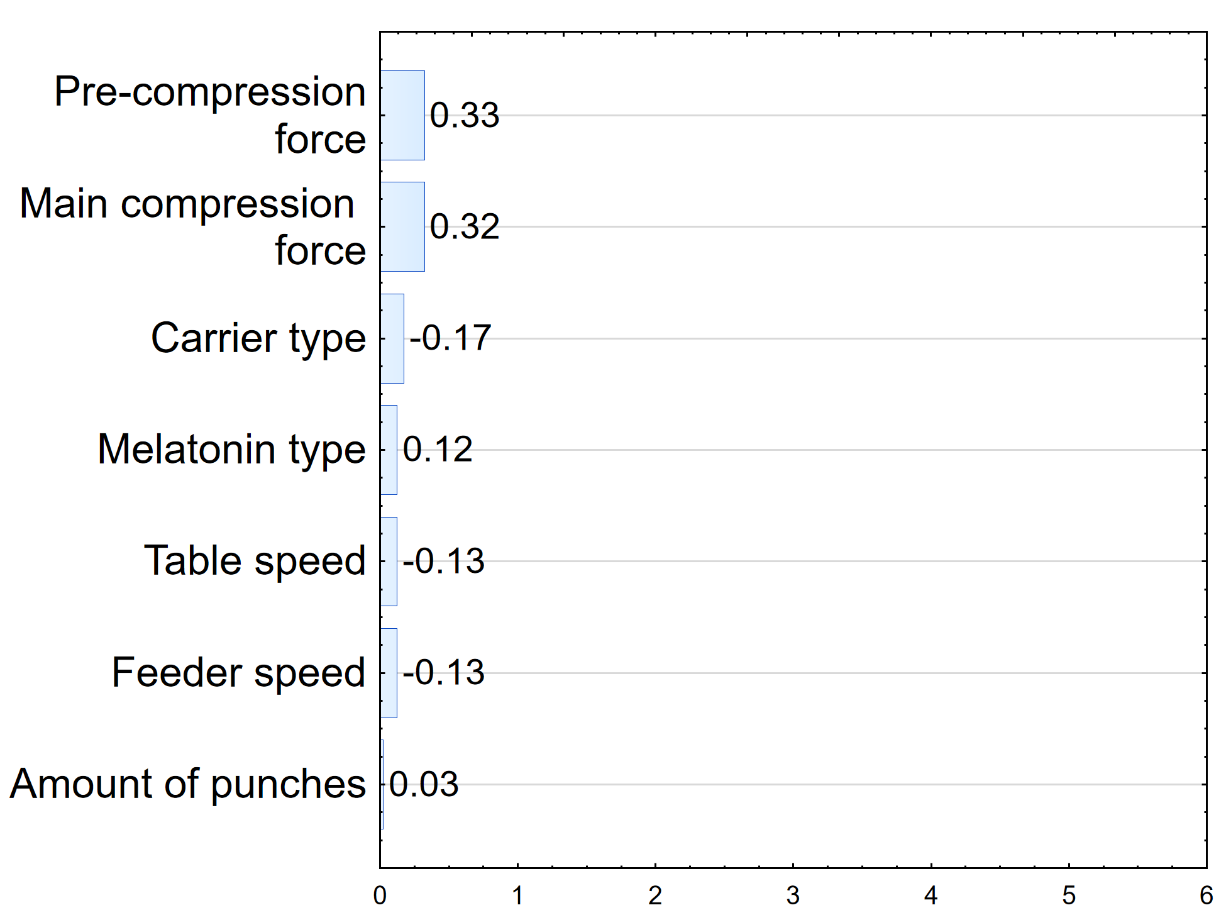
**

C – API Dissolution 15’

**
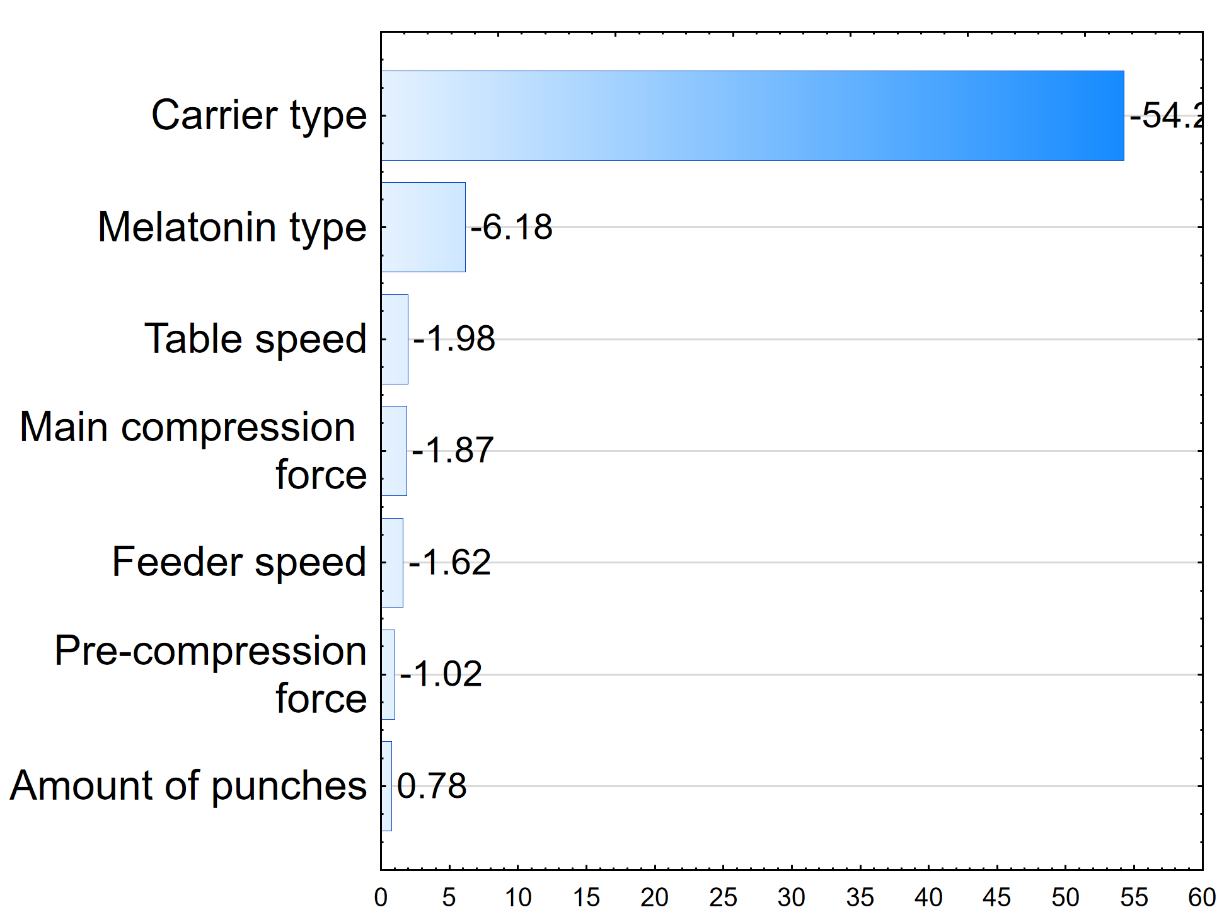
**

D – Resistance to crushing

**
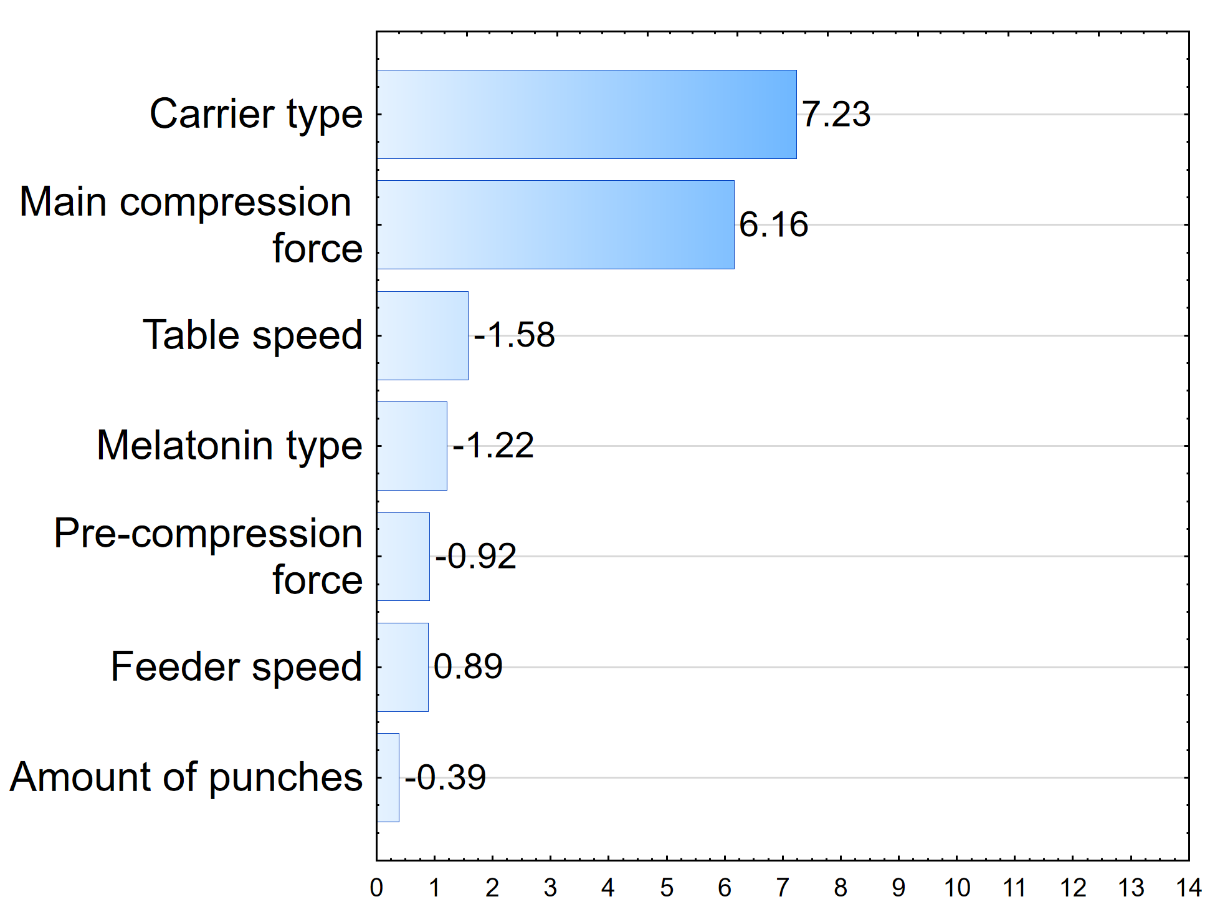
**

E – Resistance to crushing spread

**
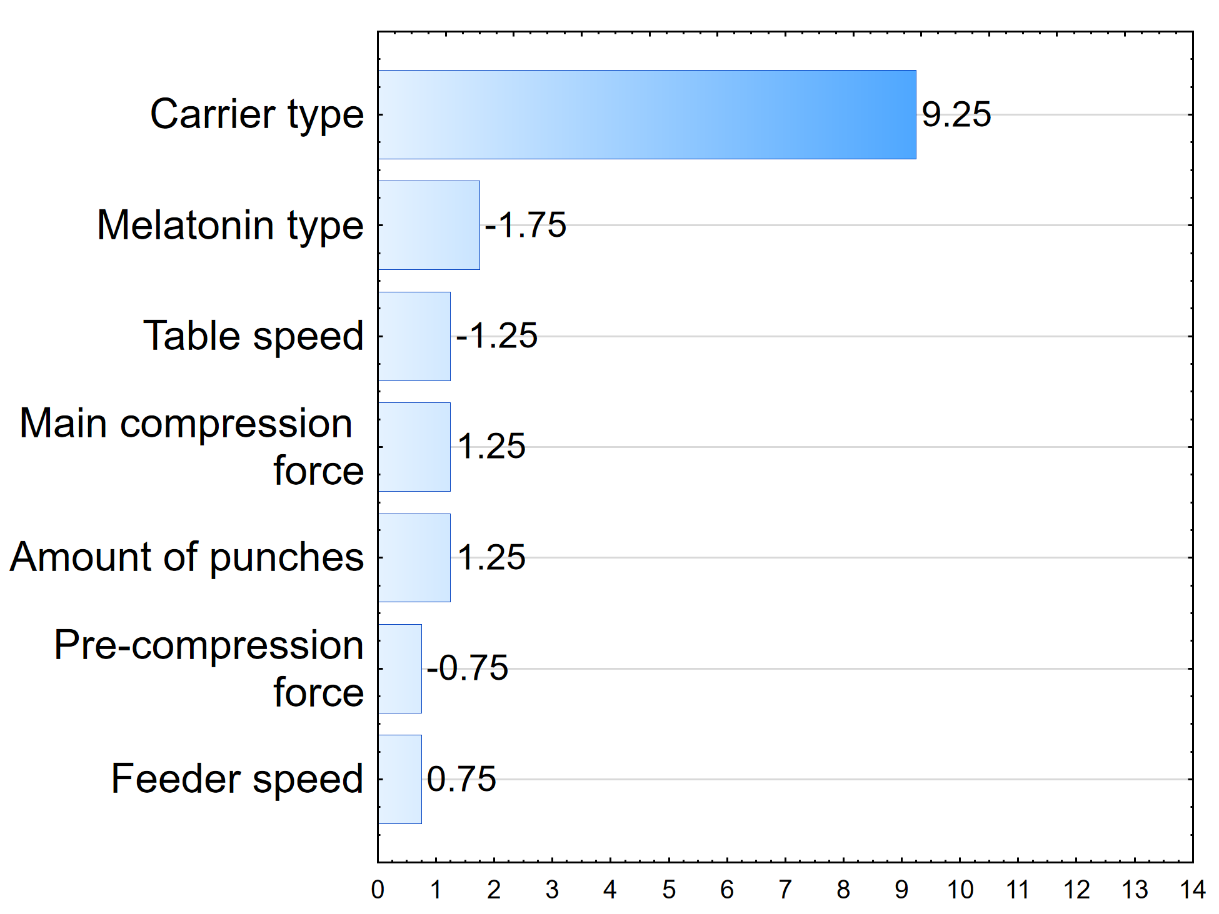
**

F – Friability

**
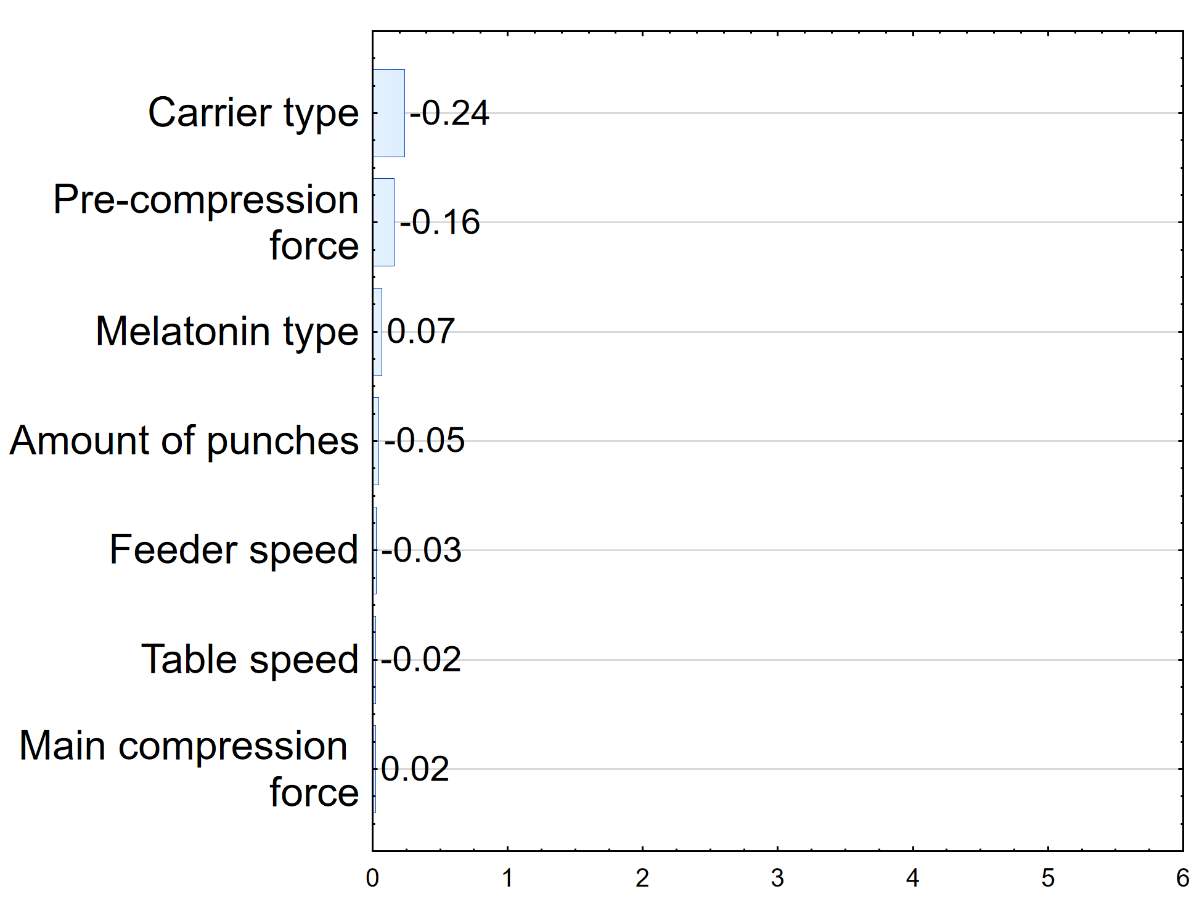
**

G – Content uniformity (AV)

**
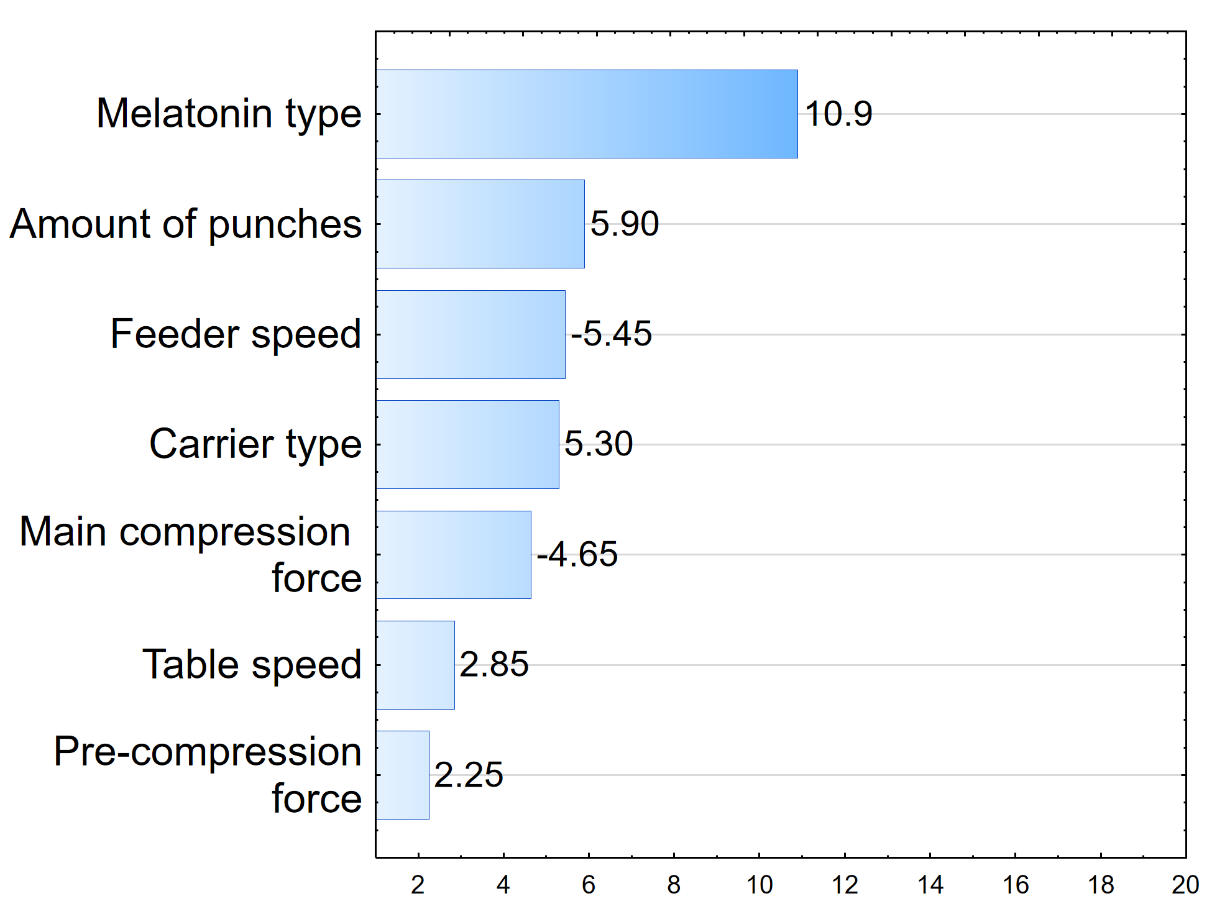
**

**Figure 3**. Residuals Shapiro-Wilk statistic (SW-W) and their p-value achieved based on full factorial design for the following models: A – Resistance to crushing, B – Resistance to crushing TXT

C – Disintegration time TXT.

| 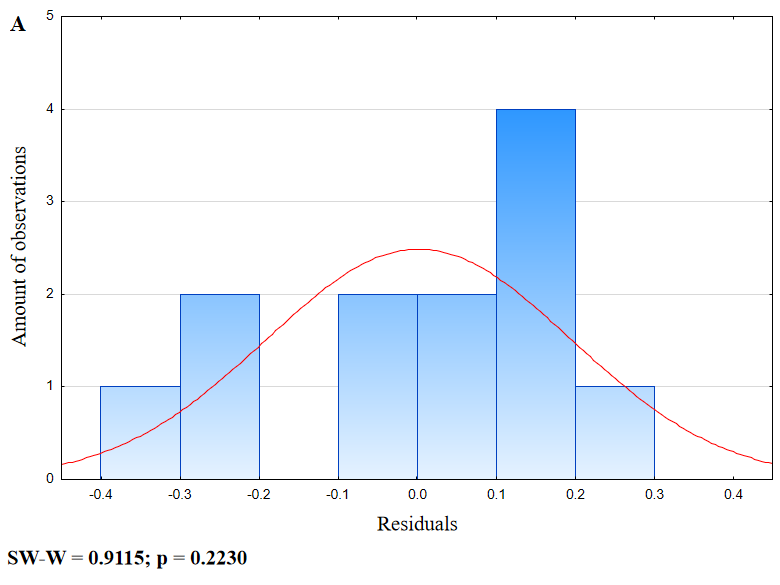 |
| --- |
| 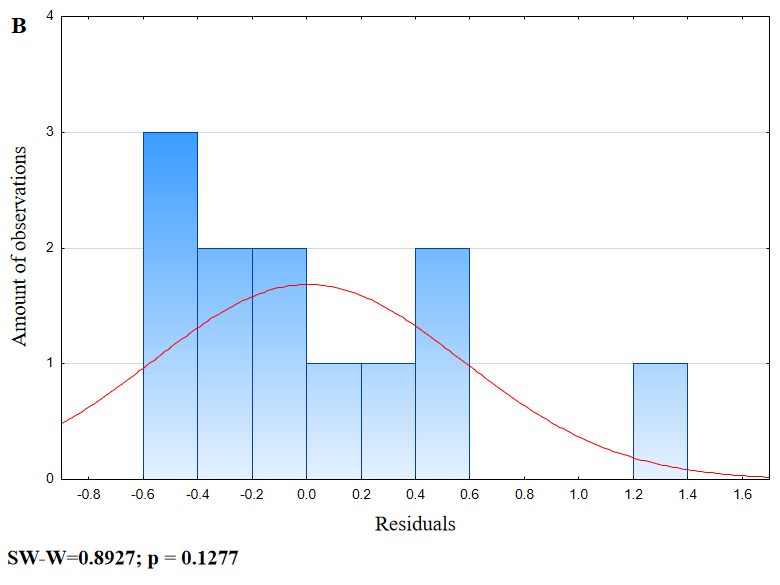 |
| 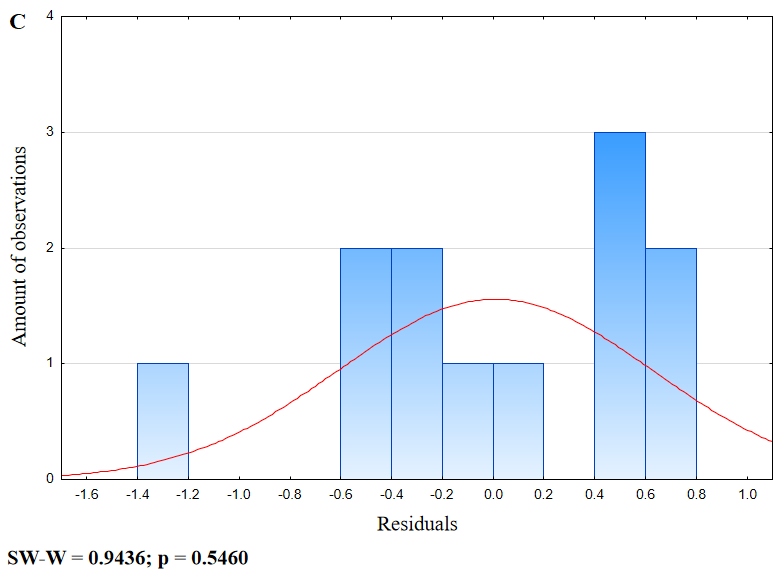 |

**Figure 4.** Residuals Shapiro-Wilk statistic (SW-W) and their p-value achieved based on fractional factorial design for Resistance to crushing TXT model.


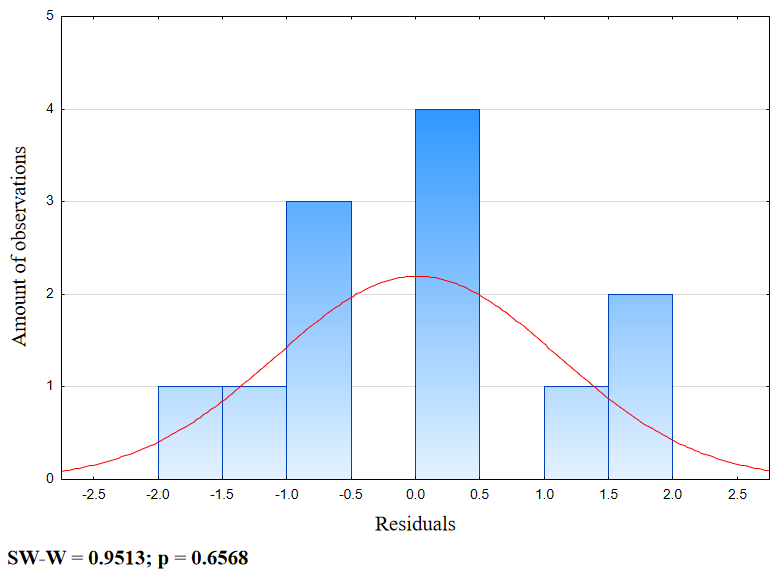


**Figure 5.** In vitro dissolution profiles of eight tested batches performed according to the Plackett-Burman design (each batch is presented as a separate run)


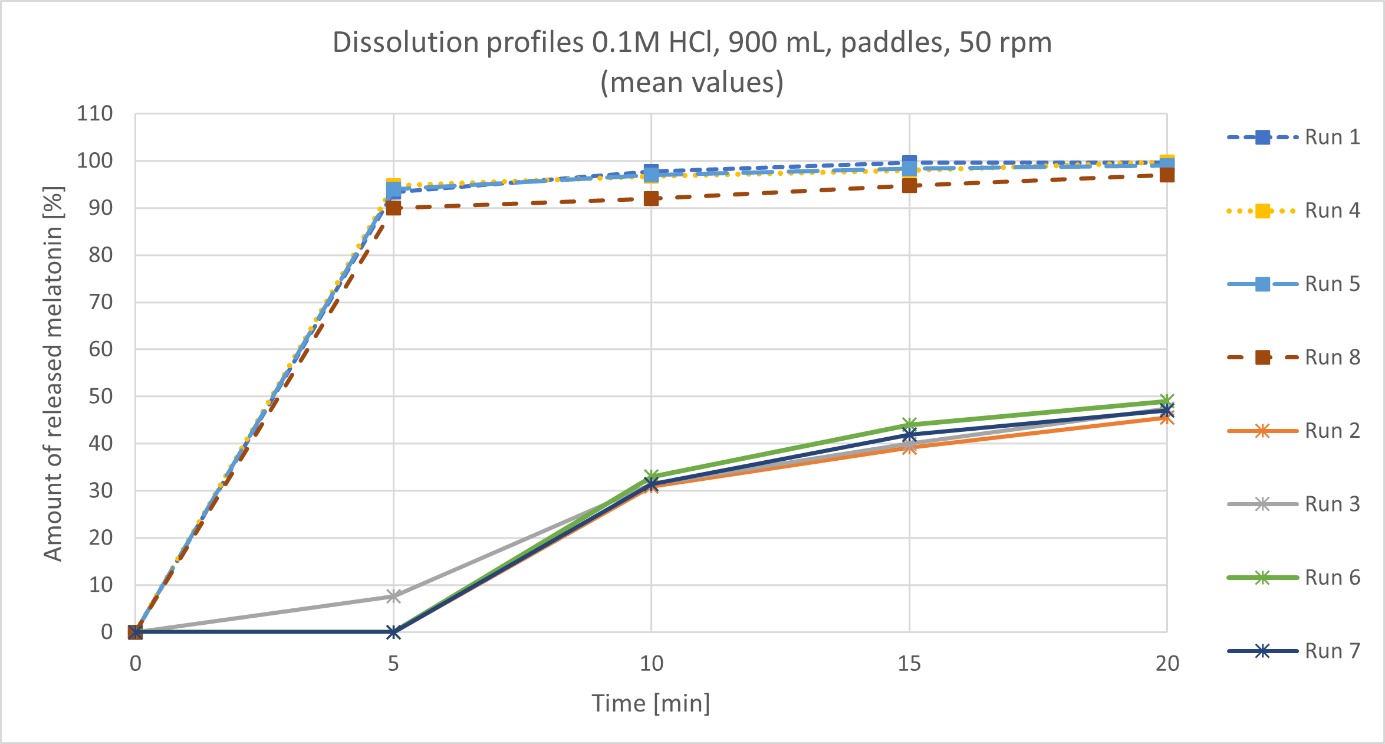


**Table I** Lack of fit statistic – full factorial design.

A – Resistance to crushing

| **Parameters** | \| **SS** \| \| --- \| | \| **df** \| \| --- \| | \| **MS** \| \| --- \| | \| **F** \| \| --- \| | **p** |
| --- | --- | --- | --- | --- | --- | --- | --- | --- | --- |
| \| **Main compression force (L)** \| \| --- \| | 7.935000 | 1 | 7.935000 | 865.6364 | 0.000086 |
| \| **Main compression force (Q)** \| \| --- \| | 0.140833 | 1 | 0.140833 | 15.3636 | 0.029532 |
| \| Lack of fit \| \| --- \| | 0.379167 | 6 | 0.063194 | 6.8939 | 0.070842 |
| \| Pure error \| \| --- \| | 0,027500 | 3 | 0.009167 |  |  |
| \| Total SS \| \| --- \| | 8,482500 | 11 |  |  |  |

B – Resistance to crushing TXT

| **Parameters** | \| **SS** \| \| --- \| | \| **df** \| \| --- \| | \| **MS** \| \| --- \| | \| **F** \| \| --- \| | \| **p** \| \| --- \| |
| --- | --- | --- | --- | --- | --- | --- | --- | --- | --- | --- |
| \| **Main compression force (L)** \| \| --- \| | 32.11066 | 1 | 32.11066 | 52.61428 | 0.005406 |
| \| **Main compression force (Q)** \| \| --- \| | 7.06603 | 1 | 7.06603 | 11.57790 | 0.042379 |
| \| Lack of fit \| \| --- \| | 1.72133 | 6 | 0.28689 | 0.47008 | 0.801925 |
| \| Pure error \| \| --- \| | 1.83091 | 3 | 0.61030 |  |  |
| \| Total SS \| \| --- \| | 42.72892 | 11 |  |  |  |

C – Disintegration time TXT

| **Parameters** | \| **SS** \| \| --- \| | \| **df** \| \| --- \| | \| **MS** \| \| --- \| | \| **F** \| \| --- \| | **p** |
| --- | --- | --- | --- | --- | --- | --- | --- | --- | --- |
| \| **Main compression force(L)** \| \| --- \| | 139.5985 | 1 | 139.5985 | 535.9197 | 0.000177 |
| \| Lack of fit \| \| --- \| | 3.3680 | 7 | 0.4811 | 1.8471 | 0.330896 |
| \| Pure error \| \| --- \| | 0.7815 | 3 | 0.2605 |  |  |
| \| Total SS \| \| --- \| | 143.7480 | 11 |  |  |  |

**Table II** Lack of fit statistic – fractional factorial design.

| **Parameters** | \| **SS** \| \| --- \| | \| **df** \| \| --- \| | \| **MS** \| \| --- \| | \| **F** \| \| --- \| | **p** |
| --- | --- | --- | --- | --- | --- | --- | --- | --- | --- |
| \| **Main compression force** (L) \| \| --- \| | 41.21675 | 1 | 41.21675 | 112.4731 | 0.001791 |
| \| **Table speed** (L) \| \| --- \| | 5.42934 | 1 | 5.42934 | 14.8157 | 0.030959 |
| \| Lack of fit \| \| --- \| | 11.95236 | 6 | 1.99206 | 5.4360 | 0.096468 |
| \| Pure error. \| \| --- \| | 1.09938 | 3 | 0.36646 |  |  |
| \| Total SS \| \| --- \| | 59.69782 | 11 |  |  |  |
